# Long-read sequencing reveals the structural complexity of genomic integration of HPV DNA in cervical cancer cell lines

**DOI:** 10.1186/s12864-024-10101-y

**Published:** 2024-02-20

**Authors:** Zhijie Wang, Chen Liu, Wanxin Liu, Xinyi Lv, Ting Hu, Fan Yang, Wenhui Yang, Liang He, Xiaoyuan Huang

**Affiliations:** 1 Department of Obstetrics and Gynecology, Tongji Hospital, Tongji Medical College, Huazhong University of Science and Technology, Wuhan, 430030 Hubei China; 2 Cancer Biology Research Center (Key Laboratory of the Ministry of Education), Tongji Hospital, Tongji Medical College, Huazhong University of Science and Technology, Wuhan, 430030 Hubei China; 3 Wuhan Kandwise Biotechnology, Inc. Wuhan, Hubei, China

**Keywords:** Cervical cancer, HPV integration, HPV16, HPV18

## Abstract

**Background:**

Cervical cancer (CC) causes more than 311,000 deaths annually worldwide. The integration of human papillomavirus (HPV) is a crucial genetic event that contributes to cervical carcinogenesis. Despite HPV DNA integration is known to disrupt the genomic architecture of both the host and viral genomes in CC, the complexity of this process remains largely unexplored.

**Results:**

In this study, we conducted whole-genome sequencing (WGS) at 55-65X coverage utilizing the PacBio long-read sequencing platform in SiHa and HeLa cells, followed by comprehensive analyses of the sequence data to elucidate the complexity of HPV integration. Firstly, our results demonstrated that PacBio long-read sequencing effectively identifies HPV integration breakpoints with comparable accuracy to targeted-capture Next-generation sequencing (NGS) methods. Secondly, we constructed detailed models of complex integrated genome structures that included both the HPV genome and nearby regions of the human genome by utilizing PacBio long-read WGS. Thirdly, our sequencing results revealed the occurrence of a wide variety of genome-wide structural variations (SVs) in SiHa and HeLa cells. Additionally, our analysis further revealed a potential correlation between changes in gene expression levels and SVs on chromosome 13 in the genome of SiHa cells.

**Conclusions:**

Using PacBio long-read sequencing, we have successfully constructed complex models illustrating HPV integrated genome structures in SiHa and HeLa cells. This accomplishment serves as a compelling demonstration of the valuable capabilities of long-read sequencing in detecting and characterizing HPV genomic integration structures within human cells. Furthermore, these findings offer critical insights into the complex process of HPV16 and HPV18 integration and their potential contribution to the development of cervical cancer.

## Background

Cervical cancer (CC) ranks fourth for both mortality and incidence among females, with 570,000 women diagnosed with CC worldwide and approximately 311,000 died from it in 2018 [1]. Human papillomavirus (HPV) is widely recognized as the primary factor contributing to CC [2, 3]. HPV is a small DNA virus with a genome consisting of an approximately 8 kb circular, double-stranded DNA molecule, which includes the early gene region (E1-E7), the long control region (LCR), and the late gene region (L1 and L2) [4–6]. HPV is the main etiological factor in the process of CC carcinogenesis [7]. However, not all HPV infections suffered by women culminate in cervical cancer [7]. A phylogenetic tree construction based on the nucleotide sequence of the L1 gene classified HPV types into five genera – alpha, beta, gamma, mu, and nu [8–13]. The Alpha genus comprises 62 HPV types that infect the mucosal epithelium [8, 10, 14, 15]. The Alpha papillomaviruses are further classified into low-risk (LR) types and high-risk (HR) types based on their potential to cause anogenital cancer [8, 10, 11, 16]. Specifically, 15 HPV genotypes have been identified as HR types, including HPV16, 18, 31, 33, 35, 39, 45, 51, 52, 56, 58, 59, 68, 73, and 82 [17]. HR HPV genotype trigger the progression from normal cells to precancerous lesions and later to invasive lesions [18]. HPV16 and HPV18, the most common high-risk types, are responsible for causing cervical diseases and together contribute to approximately 70% of cervical cancers worldwide [19, 20].

The majority of HPV infections are transient and cleared by the immune system. However, 10–20% of infections persist latently [21, 22]. Persistent HPV infection is considered the main risk factor for CC [23]. More importantly, accumulated evidence indicated that HPV DNA can integrate into human genome, which is considered as one of the most important risk factors for CC development [24–27]. For instance, Hu *et* al. reported that the rate of HPV integration increased significantly from 53.8% (14 out of 26) of cervical intraepithelial neoplasia (CIN) to 81.7% (85 out of 104) of CC cases [25]. Similarly, Huang *et* al. [26] reported that HPV integration was detected in 97.8% of CC samples and 70.5% of CIN samples with HPV infection. Additionally, they found that the incidence of HPV integration was lower for low-risk HPV types compared to high-risk HPV types in both CC and CIN samples when compared to HPV-positive normal tissues [26]. Analysis of data from 169 HPV-positive cervical cancer patients from the Cancer Genome Atlas showed that HPV integration was detected in more than 80% of patients [27]. The physical status of the HPV genome in cervical cancer could be episomal, integrated, or mixed [28–31]. Initially, HPV infects host cells in a circular form, and with persistent infection, the circular HPV genome undergoes breakage and integrates into the host genome [28–31]. During the process of HPV genome integration into the host cell genome, the E2 and/or E1 regions undergo breakage, while the long control region, and the E6 and E7 oncogenes consistently remain intact [28, 32–34]. Further studies suggest that viral integration tends to coincide with the development of high-grade cervical intraepithelial neoplasia (CIN II, III) due to the overexpression of the E6 and E7 oncogenes [35]. Measuring both the E1/E6 and/or E2/E6 ratio values is a promising prognostic tool that can offer valuable information about HPV16 integration and the physical state of HPV16 in the investigated cervical samples [36–38]. However, with the advancement of Next-Generation Sequencing (NGS) technology and targeted capture techniques, recent studies have found that breakpoints can occur anywhere in the viral genome [25–27]. Consequently, previous methods [36–38] to identify HPV16 integration status based on the E2/E6 or E1/E6 ratio may be inaccurate, as the E6 gene could be disrupted in some events.

So far, an increasing number of HPV integration sites have been identified across all chromosomes of the host cell [25, 34, 39–41]. Although These integration sites are randomly dispersed throughout the host genome, integration is not an entirely random event but also involves preferred chromosomal sites [42]. For example, HPV integration often occurring in transcriptionally active regions such as the myc oncogene [39, 43]or in chromosomally unstable regions known as common fragile sites [44, 45]. Moreover, several studies indicating that HPV16 DNA exhibits preferential sites for viral insertion, where chromosomal locations demonstrate significant homology to HPV16 DNA [25, 42, 46]. Additionally, HPV16 is observed to insert into repetitive elements scattered throughout the host chromosome, some of which are situated in close proximity to cancer-related genes [47]. However, the underlying mechanisms of HPV integration in tumorigenesis and tumor progression of CC are not well-understood. Among the various proposed mechanisms, the dysregulation of viral E6/E7 oncogene expression has been extensively studied. The integration of HPV DNA into the human genome can result in the dysregulation of the viral *E6* and *E7* oncogenes expression, which leads to the inactivation of the tumor suppressor proteins p53 and pRb, and consequent dysregulation of the apoptosis and cell cycle, respectively [48, 49]. In addition, HPV integration may also contribute to the progression of CC by promoting genomic instability or disrupting the expression and function of key host cellular genes which play vital roles in a wide range of biological processes including cell cycle regulation, cell proliferation, and apoptosis [25, 50, 51]. Previous studies have shown that HPV integration can cause amplification and rearrangement of the host cell genome, including duplications, deletions, translocations, and other events [33]. The HeLa (HPV18 integration) and SiHa (HPV16 integration) cell lines, as two classic cell line models, have been widely used in research related to HPV integration and cervical cancer. Determining the impact of HPV integration on the genome structure of these two cell lines will help further elucidate the molecular mechanisms underlying HPV integration and its role in cervical cancer. Adey *et* al. [52] conducted an exceptional and comprehensive study utilizing multiple sequencing methodologies, such as shotgun, mate-pair, and long-read sequencing, to unravel the complete genome sequence of HeLa CCL-2. The results of this study provide a high-quality reference genome that is invaluable for current and future experiments reliant on the use of HeLa cells. Akagi et al. [33] reported that HPV16 integration in SiHa cells is associated with structural variations (SVs) in nearby host genomic regions, as determined through WGS analysis. They also proposed a "looping" model, in which HPV integrant-mediated DNA replication and recombination can lead to the formation of viral-host DNA concatemers. Xu *et* al. [53] reported that HPV integration in SiHa cells causes genomic variation in the host cell genome. However, since the sequencing read length in these relevant reports for WGS analysis was relatively short, it may be difficult to accurately describe some relatively complex and longer SVs. Third-generation sequencing technologies, such as nanopore sequencing and PacBio sequencing, enable us to elucidate the complex structural aspects of genomes, due to its long read length and lack of PCR amplification, while shorter NGS reads cannot [54, 55]. It is reported that third-generation sequencing has the capability to generate extended contiguous sequences with an average read length exceeding 10 kb and subreads that can span over 60 kb [56].

Recently, HPV targeted capture-based long-read sequencing approaches have been utilized in several research studies to construct HPV genome sequences, determine integration status and sites, and explore integration patterns [57–62]. For instance, Yang *et* al. provided compelling evidence and developed a tool to demonstrate the potential usefulness of nanopore technology in identifying viral status [63]. Iden *et* al. successfully identified a total of 87 integration events, comprising 267 distinct human-HPV breakpoints in 8 tumors by PacBio long read sequencing [59]. It is worth noting that although these HPV targeted capture-based long-read sequencing approaches can effectively increase the sequencing depth of targeted sequences and reduce sequencing costs, the loss of human genomic sequence information due to targeted capture prevents a comprehensive analysis of whole-genome variations caused by HPV integration. Therefore, it is necessary to perform non-targeted capture long-read sequencing on these two cervical cancer cells to accurately reveal the complexity of the impact of HPV integration on both the HPV genome and the host cell genome structure.

In this study, we aim to precisely elucidate the genomic integration structures of HPV16 and HPV18 in CC cell lines (HeLa and SiHa) by long-read sequencing via PacBio platform. These findings provide important insights into the process of HPV16 and HPV18 integration and may provide a greater understanding of the molecular mechanisms contributing to cervical cancer.

## Results

### HPV integration breakpoints detected by targeted-capture NGS

Previous studies have utilized targeted-capture NGS for the analysis of HPV integration [25]. Our findings indicated that HPV16 integrated into chromosome 13, specifically in the intergenic region closest to the KLF5 and LINC00392 genes, in SiHa cells (Table 1). Additionally, we observed HPV18 integration into chromosome 8, specifically in the intergenic region nearest to the CCAT1 and CASC21 genes, in HeLa cells (Table 1). These results are consistent with previous findings by others [25]. However, although HPV targeted capture NGS technology has demonstrated effectiveness and cost-efficiency in detecting HPV integration, there are limitations in determining the structure of the integrated genome. Firstly, the short length of our NGS reads (150 bp) poses challenges in determining the complex intergration structure of the integrated genome. Secondly, the targeted-capture NGS strategy results in the loss of a significant portion of human genome reads, leading to an insufficient number of human reads for accurate identification of complex SVs associated with human genome. Specifically, our targeted-capture NGS reads specific to HPV16 and HPV18 represented 65.87% and 79.84% of the total sequencing reads, respectively. These findings suggest that HPV targeted NGS is primarily capable of identifying integration breakpoints between the human genome and the HPV genome, rather than providing a comprehensive understanding of the complex integrated genome structure.

**Table 1:** Summary of HPV integration sites identified using targeted-capture NGS data

Sample	Breakpoint in HPV	Integrated Position	Region	Gene
**SiHa**	HPV16: 3134	chr13: 74,087,562	intergenic	KLF5, LINC00392
	HPV16: 3384	chr13:73,788,866	intergenic	KLF5, LINC00392
**HeLa**	HPV18: 5734	chr8:128,230,629	ncRNA_intronic	CCAT1
	HPV18: 2498	chr8:128,241,546	intergenic	CCAT1, CASC21
	HPV18: 7858	chr8:128,234,255	intergenic	CCAT1, CASC21
	HPV18: 3101	chr8:128,233,367	intergenic	CCAT1, CASC21

### PacBio sequencing reveals complex HPV integrated genome structures

**Fig. 1:**
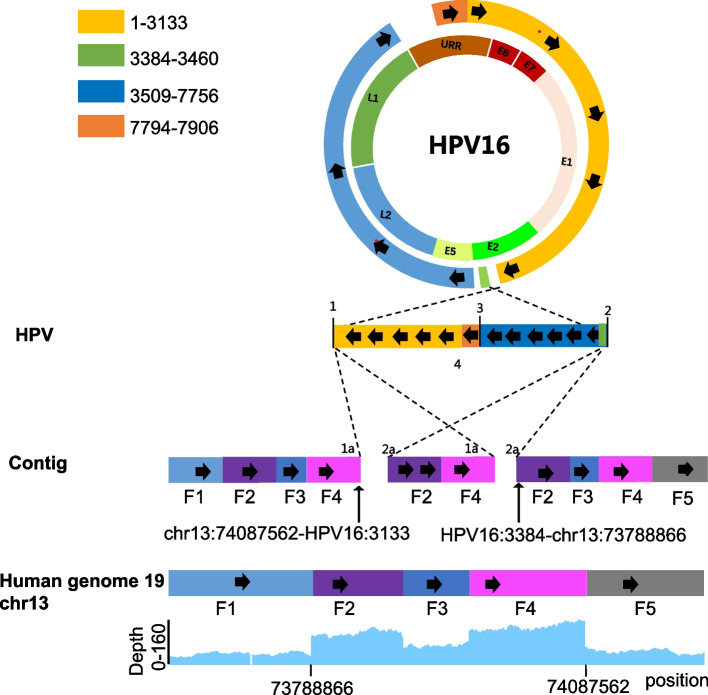
HPV16 complex integrated genome structure in SiHa cells. In the top panel, the colored regions in the outer circle represent the HPV16 sequences contained within the integrated structure, while the white regions represent the sequences that were replaced or lost as a result of integration. The inner circle shows the complete HPV16 genome for reference. The bottom panel illustrates the HPV and human reference genomes, which are connected by dotted lines to a contig that covers the integration, demonstrating how it specifically mapped to each genome(1, 2, 3, and 4 represent breakpoints on the HPV16 genome; 1a and 2a represent breakpoints on human chromosome 13; F1, F2, F3, F4, F5 represent consecutive segments on human chromosome 13). Schematic annotations of the integration event were made using data from all PacBio long reads that covered the integration event

PacBio long-read sequencing enables the generation of lengthy reads that encompass one or multiple HPV DNA fragments flanked by the human genome on both ends. This characteristic enhances the reliability and facilitates direct mapping of the reads across HPV breakpoints, thereby facilitating the elucidation of genomic integration structures. Therefore, we sequenced the genome of SiHa cells by using PacBio Sequel sequencing platform. Sequencing of SiHa cells generated a total of 194.18 Gb of bases and 8,381,208 reads after filtering out low-quality reads. The N50 read length was 34.81 kb (Table 2). Consisting with HPV targeted capture NGS results, the two integration sites: HPV16: 3134-chr13:74,087,562 and HPV16: 3384-chr13:73,788,866 were also identified by PacBio long read sequencing results in SiHa cells. These results suggesting that PacBio long-read sequencing effectively identifies HPV integration breakpoints with comparable accuracy to targeted-capture NGS methods. Furthermore, our results revealed integrated segments of the complete HPV16 L1, L2, E1, E4, E5, E6, and E7 genes, along with partial sequences of the E2 gene, within the SiHa cell genome (Fig. 1). The integration of HPV 16 occurred twice, with a fragment of HPV16 (coordinates from 3384 to 7906/1–3134) integrating into chromosome 13 at genomic coordinates 73,788,866–74,087,562 in the human genome (Fig. 1). We also observed deletions in the HPV16 genome at positions 3460–3508 and 7757–7793. Additionally, we conducted an analysis of alterations in the human genome near the site of HPV16 integration. Our findings indicate that the HPV16 fragment integrated in a reverse orientation at chr13:73,788,866 and chr13:74,087,562 (Fig. 1). Furthermore, we directly identified the chromosomal arrangement (chr13:73,255,335–73,464,522) in close proximity to the integration sites in SiHa cells (Fig. 1). Taken together, these results imply that the integration of HPV16 may contribute to the instability of the genomic structure in the vicinity of the integration site.

**Table 2:** Statistics of subreads data

	**SiHa**	**HeLa**
Subreads base(G)	194.18	211.38
Subreads number	8,381,208	7,932,747
Average subreads length	23,169	26,647
N50	34,805	40,241
Average sequencing depth	56.62	62.84
Coverage	99.04%	99.10%
≥ 4X Coverage	98.84%	98.93%
≥ 10X Coverage	98.58%	98.69%
≥ 20X Coverage	97.87%	98.15%

**Fig. 2:**
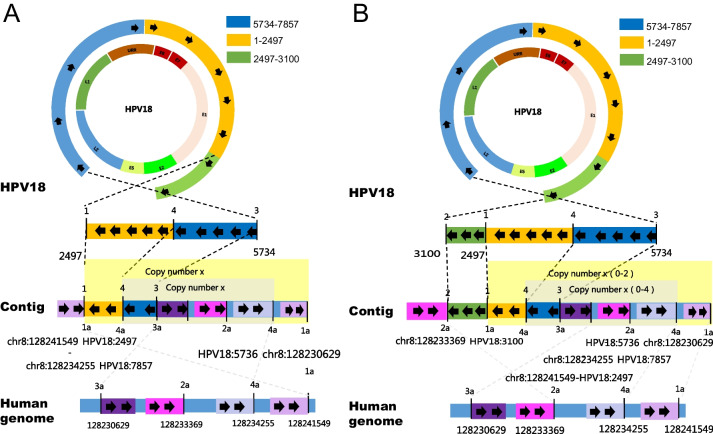
HPV18 complex integrated genome structure in HeLa cells. **A**, **B** Two proposed models of HPV18 integration genome structure in HeLa cells. In the top panel, two proposed HPV18 integration genome structures in HeLa cells are depicted. The colored regions in the outer circle represent the HPV18 sequences contained within the integrated structure, while the white regions represent the sequences that were replaced or lost as a result of integration. The inner circle shows the complete HPV18 genome for reference. The bottom panel illustrates the HPV and human reference genomes, which are connected by dotted lines to a contig that covers the integration, demonstrating how it specifically mapped to each genome. Schematic annotations of the integration event were made using data from all PacBio long reads that covered the integration event

Similarly, PacBio long-read sequencing was performed on HeLa cells. The sequencing runs of HeLa cells yielded a total of 211.38 Gb of bases and 7,932,747 reads after eliminating low-quality reads. The N50 read length was 40.24 kb (Table 2). Integration of the incomplete HPV18 genome (coordinates from 5735 to 7858/1–3100) was detected at chromosome 8q24 (Fig. 2). Integration sites identified through NGS were also confirmed by long read sequencing in HeLa cells. These results further suggest that PacBio long-read sequencing effectively identifies HPV integration breakpoints with comparable accuracy to targeted-capture NGS methods. Upon further analysis, we constructed two HPV integration models in HeLa cells based on whether the integration fragment contains HPV18: 2497–3100 (Fig. 2A and 2B). As shown in Fig. 2, a fragment of HPV18 (3101–5734) was deleted during the integration process of HPV18. Additionally, segments 1–2497, 1–3100, and 5736–7857 were identified as retro-inserted into the human genome, with the latter segment occurring in multiple copies (Fig. 2). Collectively, focal genome amplifications and rearrangements were observed in the human genome in the vicinity of the HPV18 integration site. These findings provide additional evidence that HPV integration disrupts genes near the integration breakpoints and may induce significant genomic instability, leading to genome rearrangements.

**Fig. 3:**
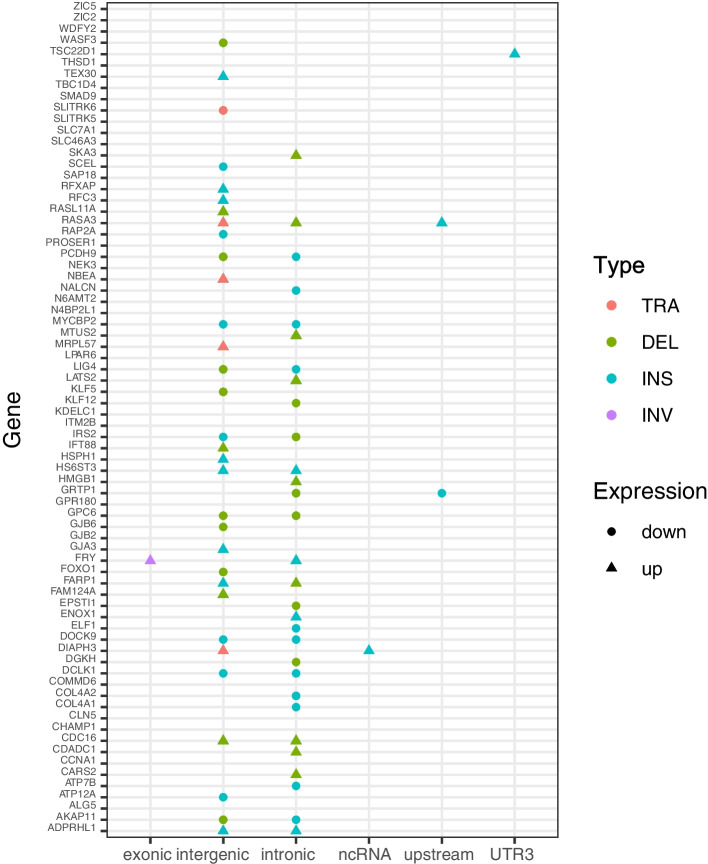
Distribution of SVs among Differentially Expressed Genes on Chromosome 13 in SiHa Cells. The x-axis represents the different regions of the genome, including exonic, splicing, ncRNA, UTR5, UTR3, intronic, upstream, downstream, and intergenic regions. The y-axis shows the differentially expressed genes. Different types of SVs are represented by different colors: TRAs (rose red), DELs (dark green), INSs (dark cyan), and INVs (pale purple). The upregulated genes are denoted by triangles, while the downregulated genes are represented by circles

PacBio long read sequencing, with its longer read lengths, can detect longer segment SVs, such as duplications (DUPs), insertions (INSs), deletions (DELs), translocations (TRAs) and inversions (INVs). Although the previous results showed that HPV integration can cause genomic SVs near integration sites, HPV integration events may also cause SVs in other regions of the human genome. Therefore, we further analyzed SVs across the whole genome in SiHa and HeLa cells, respectively. Our results showed that most of SVs were located in intergenic and intronic regions, with limited representation from 3'- or 5'-UTR, splicing, ncRNA, exonic, upstream and downstream regions (Table 3). INSs and DELs were the most common types of SVs in the human genome. Prior research has demonstrated that the integration of HPV16-LINC00393 has a significant impact on gene expression in SiHa cells [53]. Specifically, 74 genes located on chromosome 13 exhibit alterations in expression levels, with 37 genes exhibiting up-regulation and 37 genes exhibiting down-regulation [53]. SVs play a significant role in gene expression differences in humans and often affect multiple neighboring genes [64]. Therefore, to explore the potential association between changes in gene expression levels and SVs on chromosome 13 in the genome of SiHa cells, we conducted an analysis to determine whether SVs were present in the vicinity of these 74 genes. As shown in Fig. 3, our findings demonstrate that a significant proportion of genes (50 out of 74, accounting for 67.57%) exhibited SVs, indicating a potential correlation between these genomic events and alterations in gene expression patterns.

**Table 3:** Statistical analysis of SVs in SiHa and HeLa cell lines

Sample	type	exonic	splicing	ncRNA	UTR5	UTR3	intronic	Up-stream	Down-stream	intergenic
SiHa	DUP	51	1	5	0	0	13	0	1	87
	INV	87	0	21	0	1	20	1	1	117
	TRA	7	1	74	2	7	357	7	13	1460
	DEL	145	6	655	10	53	3281	75	86	8186
	INS	361	4	2273	87	281	11,823	333	256	19,282
HeLa	DUP	19	0	2	0	0	11	1	1	59
	INV	78	1	12	0	0	14	0	1	112
	TRA	9	2	71	2	6	322	6	10	1481
	DEL	135	8	814	14	64	4071	95	116	9869
	INS	349	11	2735	83	290	13,232	327	265	22,220

### Microhomologies (MHs) were identified between the HPV genome and the human genome in close proximity to the integration breakpoints

**Fig. 4:**
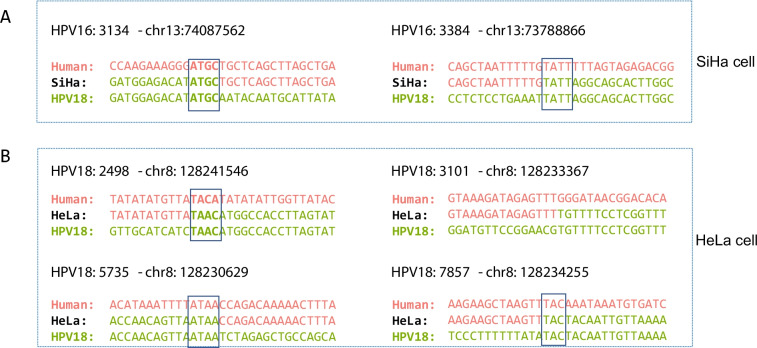
HPV integration mechanisms in SiHa and HeLa cells. **A**, **B** The figure shows the alignment of the sequence around the integration site between the human genome (rose red) and the HPV16/HPV18 genome in SiHa cells or HeLa cells (dark green), respectively. The black box represents the aligned nucleotides in the microhomology (MH) region of the two reference sequences at the HPV integration site

A previous study has indicated that microhomology-mediated recombination (MMR) could potentially serve as a mechanism for HPV integration [25]. Hence, we conducted an analysis to examine the characteristics of the HPV and human genome sequences near the integration sites in SiHa and HeLa cell lines to determine whether the integration events were associated with MMR. Two integration sites were identified in SiHa cells: HPV16: 3134-chr13:74,087,562 and HPV16: 3384-chr13:73,788,866. A microhomologous "ATGC" fragment was observed at the integration site HPV16: 3134-chr13:74,087,562. The integration site HPV16: 3384-chr13:73,788,866 exhibited a microhomologous "TATT" fragment (Fig. 4A). Four integration sites were identified in HeLa cells: HPV18: 2498-chr8: 128,241,546, HPV18: 3101-chr8: 128,233,367, HPV18: 5735-chr8: 128,230,629, and HPV18: 7857-chr8: 128,234,255. A microhomologous "TAAC/TACA" fragment was observed at the integration site HPV18: 2498-chr8: 128,241,546. The integration site HPV18: 5735-chr8: 128,230,629 exhibited a microhomologous "ATAA" fragment. At the HPV18: 7857-chr8: 128,234,255 integration site, a "TACT/TACA" fragment was observed, while no microhomologous fragment was found at the HPV18: 3101-chr8: 128,233,367 integration site (Fig. 4B). These results provide further evidence supporting the notion that MMR may indeed be the mechanism underlying HPV integration.

## Discussion

Among all cancer types, CC ranks fourth in incidence and mortality in females worldwide, with 85% of cases occurring in developing countries [1, 65]. Accumulated evidence has indicated that integration of HPV genome into the human genome is considered as one of the most important risk factors for CC development [24–27]. Although the HPV targeted-capture NGS is a highly sensitive method for detecting HPV infection and integration status, it remains challenging to accurately identify complex integrated genome structure upon HPV integration using targeted-capture NGS. Third-generation sequencing technologies, such as nanopore sequencing and PacBio sequencing, enable us to elucidate the complex structural aspects of genomes, due to their long-read length and lack of PCR amplification, whereas shorter NGS reads cannot [54, 55]. In this study, PacBio long-read sequencing was employed to accurately identify complex integrated genome structure upon HPV integration in SiHa and HeLa cell lines. We directly identified the chromosomal arrangements associated with integrated HPV16 DNA in SiHa cells (Fig. 1). Furthermore, HPV18 integration was found to induce microduplication in both HPV and human genomes (Fig. 2). HPV integration not only induces rearrangements in the chromosomes near the integration sites, but also leads to alterations within the HPV genome itself (Fig. 1–2). Specifically, as illustrated in Fig. 1, deletions were observed in the HPV16 genome at positions 3460–3508 and 7757–7793 in SiHa cells. HPV16 manifested in a concatenated repeat form. Similar outcomes were noted in HeLa cells, a fragment of HPV18 (3101–5734) was deleted during the integration process of HPV18. Additionally, segments 1–2497, 1–3100, and 5736–7857 were identified as retro-inserted into the human genome, with the latter segment occurring in multiple copies (Fig. 2). These findings are consistent with earlier reports [33, 36, 66], highlighting that HPV16 integration can trigger rearrangements within both the human and HPV genomes in cell lines and clinical samples. For example, in a study [36], extensive mapping analysis of HPV-16 E1 and E2 genes in 37 selected tumors revealed deletions in both E1 and E2 genes, with the maximum number of losses (78.4%) observed within the HPV-16 E2 hinge region. Akagi *et* al. [33]noted that HPV integrant-mediated DNA replication and recombination may lead to the formation of viral–host DNA concatemers, often disrupting genes implicated in oncogenesis and amplifying HPV oncogenes E6 and E7. Furthermore, Tsakogiannis *et* al. using the Restriction Site-PCR (RS-PCR) method, discovered two distinct rearranged HPV16 intra-viral sequences [66]. These rearrangements involve the conjunction of E2 and L1 genes, as well as the conjunction of E1 and L1 genes with inverted orientation [66]. Together, complex integrated genome structures including the HPV genome and the nearby human genome were depicted through PacBio long-read whole genome sequencing in HeLa and SiHa cells. Therefore, this study advances our understanding of the impact of HPV16 and HPV18 integration on alterations in chromosome architecture and cervical tumorigenesis.

Our results from HPV targeted-capture NGS (Table 1), along with previous studies, strongly indicate that SiHa cells have only two virus-human junctions [67, 68]. However, Diao *et* al. reported that SiHa cells contain two copies of HPV16 DNA, suggesting a possibility of four virus-human junctions[69]. These findings suggest that the two integrated HPV fragments may have the same junction and partially overlap with each other at the integration site [33]. Our PacBio long-read sequencing results have confirmed that two copies of HPV16 DNA were integrated into the human genome, which is consistent with recent findings reported by Akagi et al. [33]. Meanwhile, we identified multiple integration events in HeLa cells, and these integration events share one or two breakpoints. Additionally, some studies have shown that HPV integration can increase genomic instability [33, 70]. Therefore, we speculated that the initial integration of a single HPV fragment into a specific location in the human genome triggers increased genomic instability near the integration site, leading to subsequent focal genome amplifications and rearrangements in both the HPV and human genome sequences in the vicinity of the integration site. As a result, multiple integration events with shared breakpoints occur.

Despite the close association between HPV integration and cervical cancer development, the molecular mechanisms underlying HPV integration remain poorly understood. Previous studies have suggested that MMR is a potential mechanism for HPV integration [25]. Our findings demonstrate that two integration sites in SiHa cells and three out of the four common integration sites in HeLa cells are consistent with the mechanism of MMR, while one site deviates from this pattern. These findings indicate that the molecular mechanisms involved in HPV integration are intricate. Although MMR is a plausible molecular mechanism for HPV integration, further studies are needed to explore additional mechanisms.

Additionally, Xu *et* al. reported a significant impact of HPV16-LINC00393 integration on gene expression in SiHa cells [53]. Specifically, 74 genes located on chromosome 13 show changes in expression levels, with 37 genes up-regulated and 37 genes down-regulated [53]. Our findings revealed that the majority of these genes (50/74, 67.57%) displayed SVs in SiHa cells, indicating that integration of HPV16-LINC00393 may result in genomic SVs, thereby influencing the expression levels of associated genes (Fig. 3). Importantly, these structural variations manifest in various forms, such as DELs, INSs, DUPs, INVs, and TRAs. Furthermore, these SVs manifest in diverse regions of the human genome, encompassing UTR3, UTR5, upstream, downstream, non-coding RNA, splicing regions, exonic regions, intronic regions, and intergenic regions. Further research is required to ascertain the precise impact of these structural variations on gene expression. Meanwhile, some genes and their surrounding regions (24/74, 32.43%) may undergo expression changes in the absence of SVs, suggesting involvement of alternative regulatory mechanisms in gene expression. Elucidating these molecular mechanisms will provide additional insights into the molecular mechanisms underlying HPV integration and its association with CC.

However, this study has certain limitations. Firstly, the sample size is small, and secondly, cell lines were used instead of tissue samples. Further investigations with larger tissue sample sizes are required to explore this topic in more depth. Although tissue samples were not the primary focus of this study, Zhou *et* al. utilized another third-generation long read sequencing technology—Oxford Nanopore Technology (ONT) sequencing—to investigate HPV integration in clinical samples [57]. Notably, ONT sequencing revealed that HPV16 integration tends to induce genomic instability, rearrangements and SVs near integration sites [57]. This aligns with the conclusions drawn from our cell line samples, reinforcing the findings obtained through PacBio sequencing in cell lines. While ONT sequencing was employed in the investigation of HPV integration, our research utilized PacBio sequencing. Both methods belong to third-generation sequencing technologies, leveraging their extended read lengths to detect intricate genomic structures. It is crucial to acknowledge that each technology, including ONT and PacBio, has its own set of advantages and disadvantages. On the one hand, ONT sequencing generates longer contiguous sequence reads, with higher throughput, portability, and lower cost, making ONT promising for various applications, especially for genome-wide and transcriptome-wide studies requiring large amounts of data [71]. On the other hand, PacBio can produce higher quality raw data with a lower error rate and higher mappability compared to ONT raw data, proving invaluable for dissecting intricate genomic features, uncovering SVs, and elucidating epigenetic modifications [71, 72]. The selection of sequencing technology should be tailored to meet the specific needs of our study. Thus, each of these two technologies has its own strengths and weaknesses and should not be used interchangeably in different studies.

## Conclusions

In this study, we have successfully depicted high-resolution complex models of HPV integrated genome structures, specifically HPV16 and HPV18, in SiHa and HeLa cell lines using PacBio long-read sequencing. This achievement serves as a compelling demonstration of the invaluable capabilities of long-read sequencing in the detection and characterization of viral genomic integration structures within host cells. Moreover, these findings offer significant insights into the intricate process of HPV16 and HPV18 integration, thereby contributing to a better understanding of the molecular mechanisms underlying cervical cancer development.

## Methods and materials

### Cell culture

HeLa and SiHa cell lines were obtained from the American Type Culture Collection (Manassas, VA, USA). Two cell lines were cultured in DMEM medium (Gibco) supplemented with 1% streptomycin / penicillin solution (Gibco) and 10% fetal bovine serum (Gibco). The cell lines were maintained in an incubator with 5% CO_2_ at 37 °C.

### Targeted-hybridization short-read sequencing

The Genomic DNA Extraction Kit (Vazyme, Nanjing, China) was used to extract genomic DNA according to the instructions of the manufacturer. The DNA concentration was quantified using the Qubit dsDNA HS Assay Kit (Thermo Fisher). 500 ng of DNA was divided into fragments (200 bp to 300 bp) by Enzymes (Vazyme, Nanjing, China), then these fragments were end-repaired, dA-tailed, and adaptor ligated. ligation products were PCR-amplified and obtained a DNA library. The concentration of the DNA library was quantified using a Qubit dsDNA HS Assay Kit. Hybridization was performed using the HPV probes for the full-length HPV16/18 genomes and a GenCap enrichment kit according to the Target Enrichment Protocol (iGeneTech, Beijing, China). In brief, 500 ng of DNA library were hybridized with HPV probes at 65 °C for 16 h, and the washing buffer was used to remove un-captured fragments. These enriched fragments were amplified by PCR. The hybridization procedure was conducted for twice. Lastly, the enriched libraries were sequenced on an Illumina Sequencer (HiSeq 2000) with 150 bp paired-end reads.

### PacBio long-read sequencing

Genomic DNA of HeLa and SiHa cancer cell lines were extracted using the Genomic DNA Extraction Kit (Vazyme, Nanjing, China). DNA integrity was checked with the Agilent 4200 Bioanalyzer (Agilent Technologies). A total of 8 μg genomic DNA was sheared using Covaris g-TUBEs (Covaris) and then purified using AMPure PB magnetic beads. SMRT bell libraries were prepared with the SMRTbell Express Template Prep Kit 2.0 (Pacific Biosciences). Each library was size-selected on a BluePippin system for 25 kb molecules, followed by annealing the sequencing primer and the binding polymerase to SMRTbell templates. Libraries were then sequenced on Pacific Bioscience Sequel II platform (Pacific Biosciences) at Frasergen Bioinformatics Co., Ltd (Wuhan, China).

### Short-read sequence alignment

The targeted-capture NGS data was used to determine the HPV integration status present in samples. Firstly, clean reads were obtained by removing adaptor-contaminated reads, duplicated reads and low-quality reads. Clean reads were aligned to the human genome (GRCh38) and HPV genome (HPV16: NC_001526.4, https://www.ncbi.nlm.nih.gov/nuccore/NC_001526.4/; HPV18: AY262282.1, https://www.ncbi.nlm.nih.gov/nuccore/AY262282.1) using a Burrows–Wheeler Aligner (BWA) [73]. Subsequently, the paired-end reads that aligned perfectly to HPV or human reference genome were removed. Lastly, the human-HPV chimeric paired-end reads were used to define the position of breakpoints in the human genome and HPV genome.

### Long-read sequence alignment

The subreads sequences were obtained by processing the raw sequence data on SMRTlink v9.0 software. The clean reads were mapped to a merged reference genome that comprises human genome (GRCh38) and HPV genome (HPV16: NC_001526.4, HPV18: AY262282.1) with a long-read mapper: Minimap2 (v2.24; https://github.com/lh3/minimap2) [74]. Output sequence alignment/map (SAM) files were transformed to binary format (BAM) files, and then were sorted and indexed with samtools (v1.13; http://samtools.sourceforge.net/) [75]. Then Sniffles software was used to call SVs from the bam file. ANNOVAR was used to do the annotation for the integrated breakpoints [76].

## Data Availability

The sequencing raw data have been deposited in the Genome Sequence Archive of the National Genomics Data Center, China National Center for Bioinformation, under accession number HRA007085 (https://ngdc.cncb.ac.cn/).
